# Benign Fibrous Histiocytoma of the Maxilla: A Rare Case and Updated Review

**DOI:** 10.7759/cureus.97724

**Published:** 2025-11-25

**Authors:** Subhasish Burman, Asish K Das, Moumita Ghosh, Abhishek Khatua, Diptangshu Mallick

**Affiliations:** 1 Oral and Maxillofacial Surgery, Dr. R. Ahmed Dental College and Hospital, Kolkata, IND

**Keywords:** benign fibrous histiocytoma, jaw tumor, maxilla, oral cavity, soft tissue neoplasm

## Abstract

Benign fibrous histiocytoma (BFH) is a soft tissue neoplasm composed of both fibroblast and histiocyte cells. These lesions mostly occur in soft tissue and can present as a fibrous mass anywhere in the body. The involvement of the jawbone is extremely rare. The etiology of this entity remains obscure. Here, we report a case of BFH involving the maxilla. The clinicopathological features of this tumor involving the maxilla have been discussed with a brief literature review of this pathology involving the maxilla.

## Introduction

Benign fibrous histiocytoma (BFH) is a soft tissue neoplasm originating from mesenchymal cells and most commonly found in the skin of the extremities. It was first described by Stout and Lattes in 1967. It mostly occurs in the soft tissue and rarely involves bone, accounting for approximately 1% of all benign bone tumors [1]. The most frequently involved bones are the femur, tibia, and pelvic bone. Involvement of the jawbone is extremely rare, and very few cases have been reported in the literature to date [2]. Only four cases of BFH involving the maxilla have been reported in the literature. However, a few cases of malignant tumors have also been reported. Here, we report a rare case of BFH involving the maxilla in a middle-aged adult male patient who reported to our Department of Oral and Maxillofacial Surgery with a complaint of a right-sided facial swelling and was treated in our institution.

## Case presentation

A 48-year-old male patient presented to the Department of Oral and Maxillofacial Surgery at Dr. R. Ahmed Dental College and Hospital, Kolkata, India, with a chief complaint of swelling on the right side of the face for one year. He gave a history of trauma on the same side of his face one month back, following which he developed pain in the upper left posterior teeth region. He consulted a dentist and underwent teeth extraction in the same region. After extraction, he developed swelling on the right side of his face, which gradually increased in size and was not associated with any pain. There was no history of pus discharge or bleeding in the associated region. He also complained of nasal obstruction on the right side and epiphora from the right eye for the past six months. His past medical and family history was non-contributory, and the rest of the general physical examination showed no other abnormality.

**Figure 1:**
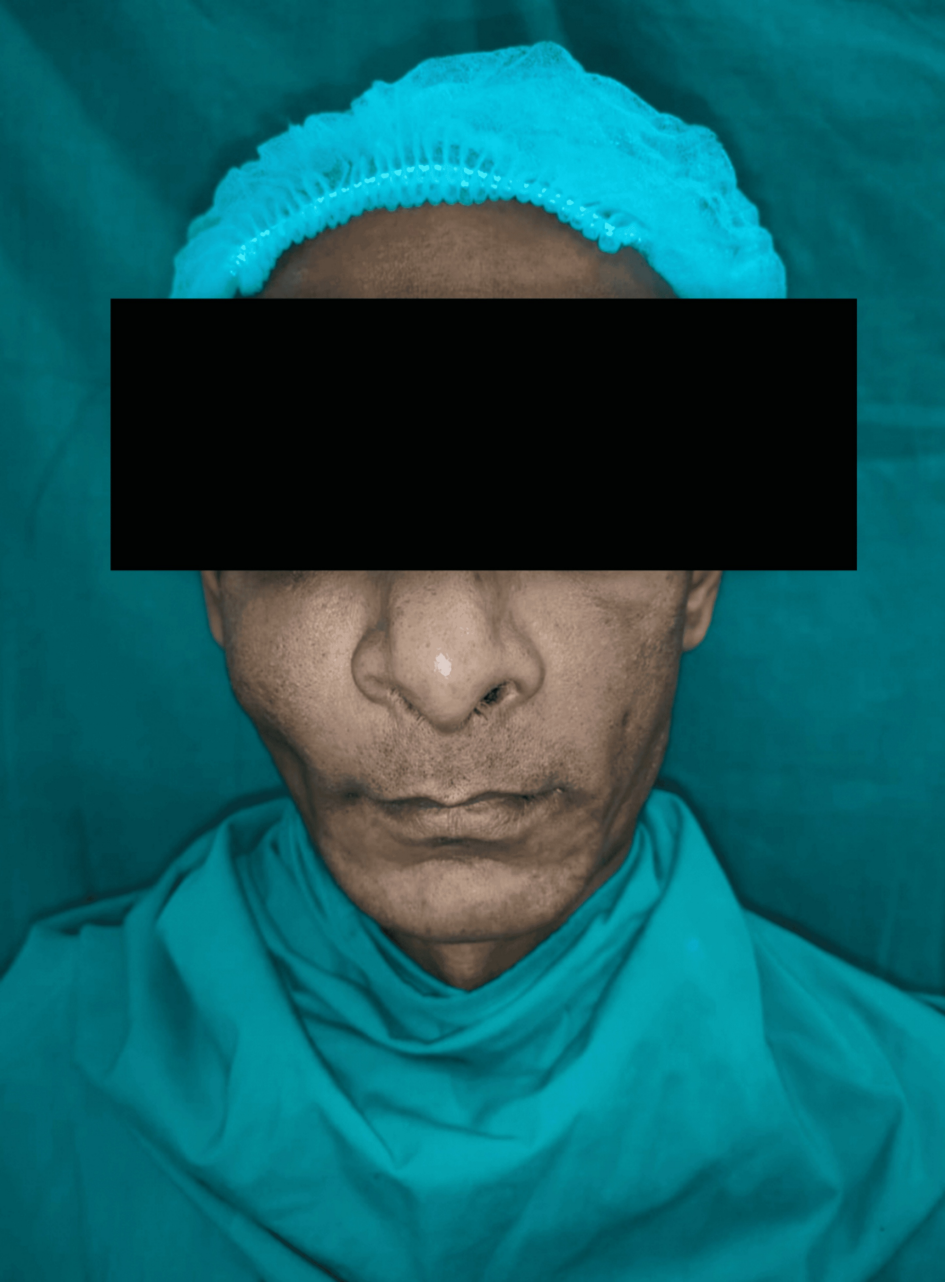
Extraoral photograph of the patient showing mild asymmetry on the right half of the face.

**Figure 2:**
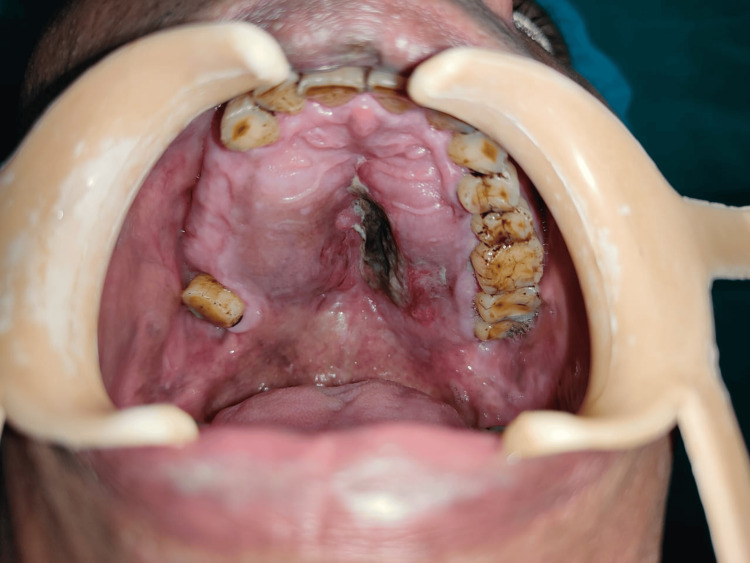
Intraoral view showing bilateral palatal expansion and black pigmentation in the mid palatal region.

Extraoral examination revealed mild facial asymmetry and the presence of diffuse swelling over the middle third of the face on the right side extending from the right lower eyelid to the ala of the nose superoinferiorly (Figure 1). On palpation, a hard, non-tender, non-compressible, non-fluctuant swelling measuring approximately 5 cm × 4 cm in its greatest dimension was evident. Other clinical features, such as a decrease in right eye palpebral fissure width and a deviated nasal septum to the left side, were seen. Ocular examination revealed normal visual acuity and normal extraocular muscle movement. Intraoral examination revealed bilateral palatal swelling involving the entire hard palate. Multiple posterior teeth were missing on the right side of the maxilla. A non-scrapable, hyperpigmented patch encircled by an erythematous region was noticed over the left side of the hard palate near the midline. The rest of the overlying mucosa was normal in color and texture (Figure 2). The swelling was hard on palpation and non-tender. No existing teeth were mobile, and there was no evidence of discharge or any other associated symptoms.

**Figure 3:**
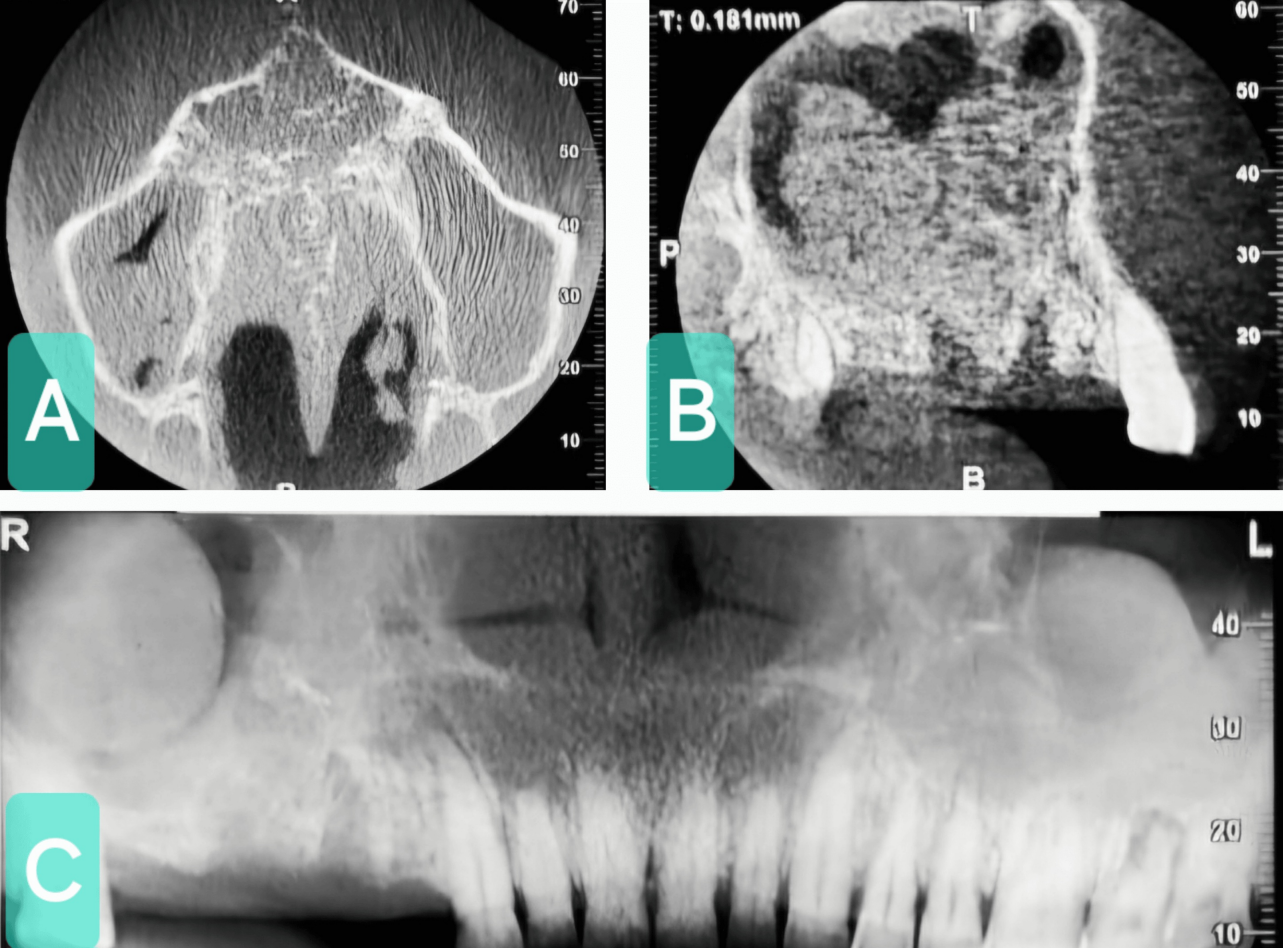
Cone-bean CT scan. (A) Axial view. (B) Sagittal view. (C) A well-defined soft tissue mass can be seen within the maxillary antrum.

Radiographic examination included cone-beam CT (CBCT) and CT scans. The CBCT report revealed the presence of a well-defined soft tissue density mass in both maxillary sinuses (Figure 3). The CT scan report revealed a right upper alveolobuccal mass with extension 5.2 cm × 4.8 cm × 3 cm (superoinferior × mediolateral × anteroposterior) and evidence of erosion of palatal bone. The nasal septum was deviated to the left side with hypertrophy of the right inferior turbinate. An enlarged cervical lymph node on the right side of the neck at levels IIA and IIB was noted. Radiological investigations such as CT and CBCT helped assess the extent of the lesion and determine any bony changes. Ultrasound-guided fine-needle aspiration cytology of the lymph nodes revealed reactive hyperplasia of the lymph nodes. No evidence of malignancy was noted. Based on clinical and radiological evaluation, a provisional diagnosis of maxillary antral polyp and differential diagnosis of sinonasal tumor, calcifying epithelial odontogenic tumor, ossifying fibroma, and calcifying odontogenic cyst was made.

**Figure 4:**
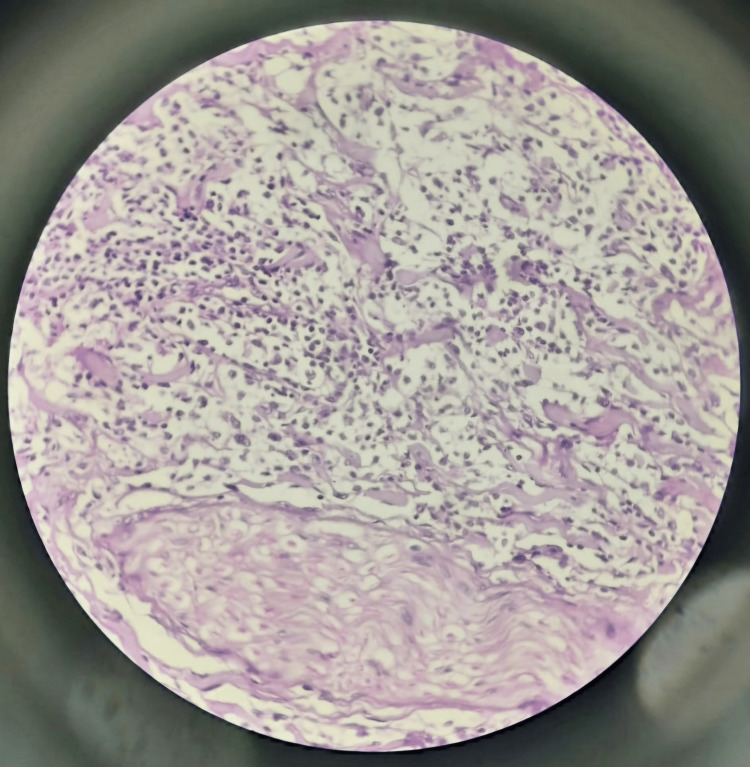
Hematoxylin and eosin-stained section showing a storiform pattern arrangement of spindle-shaped cells (×100).

An incisional biopsy was advised under local anesthesia, and a hematoxylin and eosin-stained section revealed the presence of an area showing dense spindle cell proliferation with varying degrees of collagenization, with the presence of interfacing fascicles of spindle cells arranged in a storiform pattern (Figure 4). Immunohistochemistry (IHC) was advised for confirmatory diagnosis. IHC staining showed S-100, CD68, STAT6, and CD34 negativity. Negativity for S-100 showed that the lesion could be differentiated from leiomyosarcoma and neurogenic tumors. The histological and IHC features were in keeping with BFH.

**Figure 5:**
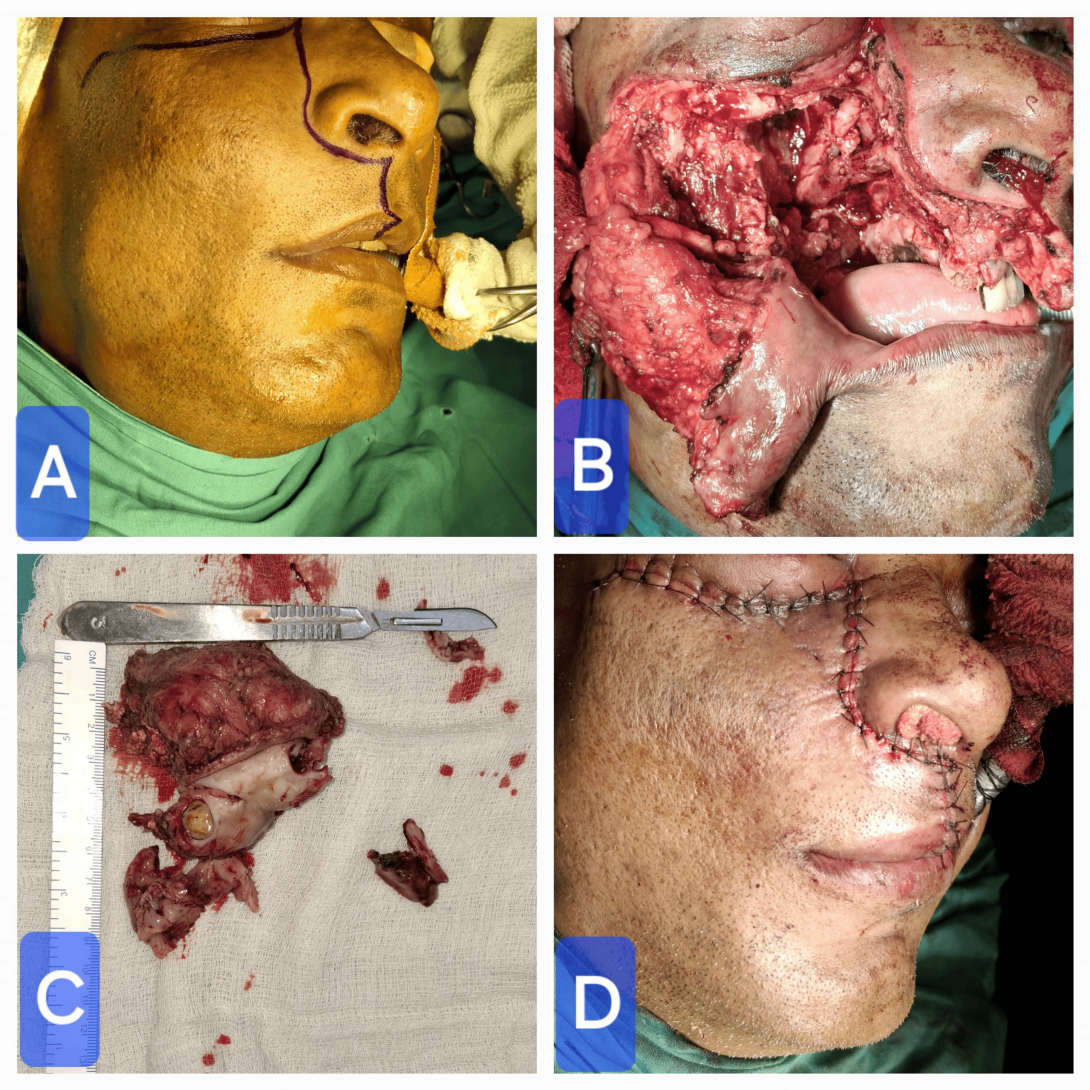
Intraoperative images. (A) Weber Ferguson incision with Dieffenbach modification. (B) Subtotal maxillectomy. (C) Gross specimen of the right maxilla after subtotal maxillectomy. (D) Layer-by-layer closure of the incision.

As BFH is locally aggressive, and in our case, erosion of the bone was noted, the treatment planned was a subtotal maxillectomy under general anesthesia. Weber Ferguson incision with Dieffenbach modification was performed for exposing the right maxilla. A subtotal maxillectomy preserving the orbital floor was done using surgical burs, osteotome, and mallet. Layer-by-layer closure was done using 3-0 Vicryl, and skin suture was made using 3-0 Prolene (Figure 5). Postoperatively, an interim obturator was given, and the patient was kept under a Ryle’s tube feeding for four weeks. The final histopathology report confirmed BFH. The patient has been followed up periodically and is doing well as of the fourth month postoperatively.

## Discussion

BFH is a soft tissue neoplasm consisting of histiocytes and fibroblasts. It was first described by Stout and Lattes in 1967 [1,2]. It commonly occurs in sun-exposed skin regions, especially the extremities, but rarely involves bones of the head and neck region. According to the WHO histological classification of tumors, BFH is defined as a benign lesion with rare mitosis and absence of cellular atypia and composed of spindle-shaped fibroblasts arranged in a unique storiform pattern with variable admixture of small, multinucleated, osteoclast-like giant cells. Foamy cells (xanthoma), chronic inflammatory cells, stromal hemorrhages, and hemosiderin pigment are also commonly present [3-6].

To date, fewer than 100 cases have been reported according to the WHO [7,8]. BFH affecting the bone is very rare and comprises fewer than 1% of all benign bone tumors [7]. Of note, the head and neck region is a relatively rare site for BFH, and to date, very few cases have been reported. Only 10 cases of fibrous histiocytoma in the maxilla have been reported in the literature, which includes both malignant and benign variants, and only eight cases of BFH involving the neurocranium have been reported (Table 1). The etiology of the lesion is not yet clear. From a review of the literature, most reported cases were in the age group of 10-50 years, with a mean age of 40 years and a male predilection (2.5:1) [5,6]. We reported the case of a 48-year-old male patient whose right maxilla was involved. Clinically, they present as a slow-growing, painless tumor. CBCT and CT scans should be requested to detect the extent of bony involvement, as it helps in treatment planning. The confirmatory diagnosis can be made based on histopathology and IHC study. IHC plays a vital role in the diagnosis of BFH. The prognosis of oral BFH is very good, and the mainstay of treatment is surgical en bloc resection of the tumor with a safe margin of 5 mm [7]. Although malignant variants have also been reported in the literature, the incidence of BFH to malignant transformation is very rare at 1% [9-14].

**Table 1:** A review of fibrous histiocytoma cases in the maxilla. BFH = benign fibrous histiocytoma; MFH = malignant fibrous histiocytoma

Authors	Number of cases	Benign/Malignant variant	Age/Sex	Location	Treatment modality	Year
Cale et al. [1]	1	BFH	13 years/Male	Maxilla	Surgical excision	1989
Saluja et al. [3]	1	BFH	23 years/Female	Maxilla	Surgical excision	2014
Besly et al. [8]	2	MFH	-	Maxilla	Surgical excision	1993
Shahoon et al. [9]	1	MFH	36 years/Male	Maxilla	Surgical excision	2001–2009
Kasat et al. [10]	1	MFH	46 years/Female	Maxilla, hard palate	Surgical excision	2014
Mohanty et al. [5]	1	BFH	24 years/Male	Posterior maxilla	Surgical excision	2020
Singh Chauhan et al. [7]	1	BFH	45 years/Male	Maxilla (maxillary sinus)	Surgical excision	2020
Sharma et al. [12]	1	BFH	30 years	Maxilla (anterior maxilla)	Surgical excision	2015
Soman et al. [14]	1	BFH	29 years/Male	Maxilla	Surgical excision	2021

Radiological investigations such as CBCT and CT assess bony changes such as expansion and erosion, helping in accurate diagnosis [15]. Biopsy and IHC act as useful aids for confirming the diagnosis. In our case, as the tumor had eroded a part of the maxillary bone, we opted for subtotal maxillectomy to prevent any future recurrence. Because of its locally aggressive nature, a long-term follow-up is required.

## Conclusions

Oral BFH tumors generally have an excellent prognosis and a low risk of recurrence. However, due to their locally aggressive nature, complete surgical resection is the primary treatment approach. Clinically, it can be challenging to differentiate BFH from other soft tissue neoplasms. Therefore, histopathological and IHC staining, along with radiological correlation, are required for a confirmatory diagnosis. Chemotherapy or radiotherapy has no role in the management of the benign variant of fibrous histiocytoma. There is a low risk of recurrence with BFH if complete excision of the lesion is performed with wide margins. As with any lesion, long-term follow-up is necessary to monitor for tumor recurrence.
